# Dicyclo­hexyl­ammonium bis­(chloro­difluoro­acetato-κ*O*)cyclo­hexyl­diphenyl­stannate(IV)

**DOI:** 10.1107/S1600536808011057

**Published:** 2008-04-26

**Authors:** Yin Yin Teo, Kong Mun Lo, Seik Weng Ng

**Affiliations:** a Department of Chemistry, University of Malaya, 50603 Kuala Lumpur, Malaysia

## Abstract

In the title mixed-organyl stannate, (C_12_H_24_N)[Sn(C_6_H_5_)_2_(C_6_H_11_)(CClF_2_O_2_)_2_], there are two cations and two anions in the asymmetric unit. Each five-coordinate Sn atom shows *trans*-C_3_SnO_2_ trigonal bipyramidal coordination. The four Sn—O distances are approximately equal in the two independent anions. Each ammonium cation serves as a hydrogen-bond donor to two stannates, the hydrogen-bonding inter­actions giving rise to linear hydrogen-bonded chains.

## Related literature

For the structure of the bis­(chloro­difluoro­acetato)cyclo­hexyl­diphenyl­stannate ion, see: Teo *et al.* (2007 ▶). For other dicyclo­hexyl­ammonium di(carboxyl­ato)triorganostannates, see: Ng & Hook (1999 ▶); Ng & Kumar Das (1992 ▶; 1993 ▶); Ng & Rae (2000 ▶); Ng *et al.* (1990 ▶; 1991*a* ▶,*b* ▶,*c* ▶; 1992 ▶; 2000 ▶). For a review of the structural chemistry of organotin carboxyl­ates, see: Tiekink (1991 ▶, 1994 ▶).
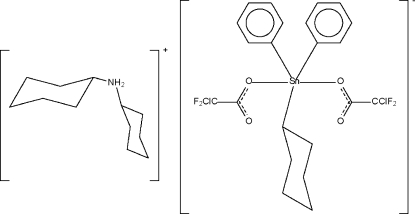


## Experimental

### 

#### Crystal data

(C_12_H_24_N)[Sn(C_6_H_5_)_2_(C_6_H_11_)(CClF_2_O_2_)_2_]
*M* *_r_* = 796.29
Monoclinic, 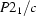

*a* = 22.5469 (3) Å
*b* = 17.2799 (2) Å
*c* = 18.6963 (2) Å
β = 90.324 (1)°
*V* = 7284 (2) Å^3^
*Z* = 8
Mo *K*α radiation
μ = 0.91 mm^−1^
*T* = 100 (2) K
0.15 × 0.15 × 0.10 mm

#### Data collection

Bruker SMART APEX diffractometer
Absorption correction: multi-scan (*SADABS*; Sheldrick, 1996 ▶) *T*_min_ = 0.788, *T*_max_ = 0.915
93218 measured reflections
16710 independent reflections
12483 reflections with *I* > 2σ(*I*)
*R*_int_ = 0.033

#### Refinement

*R*[*F* ^2^ > 2σ(*F* ^2^)] = 0.066
*wR*(*F* ^2^) = 0.216
*S* = 1.33
16710 reflections
829 parameters
240 restraints
H-atom parameters constrained
Δρ_max_ = 2.24 e Å^−3^
Δρ_min_ = −2.03 e Å^−3^

### 

Data collection: *APEX2* (Bruker, 2007 ▶); cell refinement: *SAINT* (Bruker, 2007 ▶); data reduction: *SAINT*; program(s) used to solve structure: *SHELXS97* (Sheldrick, 2008 ▶); program(s) used to refine structure: *SHELXL97* (Sheldrick, 2008 ▶); molecular graphics: *X-SEED* (Barbour, 2001 ▶); software used to prepare material for publication: *publCIF* (Westrip, 2008 ▶).

## Supplementary Material

Crystal structure: contains datablocks global, I. DOI: 10.1107/S1600536808011057/tk2258sup1.cif

Structure factors: contains datablocks I. DOI: 10.1107/S1600536808011057/tk2258Isup2.hkl

Additional supplementary materials:  crystallographic information; 3D view; checkCIF report

## Figures and Tables

**Table:** 

Sn1—C1	2.144 (6)
Sn1—C7	2.125 (6)
Sn1—C13	2.134 (6)
Sn1—O1	2.226 (4)
Sn1—O3	2.231 (4)
Sn2—C23	2.145 (5)
Sn2—C29	2.130 (5)
Sn2—C35	2.138 (6)
Sn2—O5	2.243 (4)
Sn2—O7	2.263 (4)

**Table:** 

O1—Sn1—O3	177.3 (2)
O5—Sn2—O7	178.8 (2)
C19—O1—Sn1	121.2 (3)
C21—O3—Sn1	128.9 (4)
C41—O5—Sn2	134.4 (4)
C43—O7—Sn2	124.4 (3)
C45—N1—C51	116.8 (6)
C57—N2—C63	118.3 (5)

**Table 2:** Hydrogen-bond geometry (Å, °)

*D*—H⋯*A*	*D*—H	H⋯*A*	*D*⋯*A*	*D*—H⋯*A*
N1—H1n1⋯O2	0.88	1.93	2.797 (7)	166
N1—H1n2⋯O6^i^	0.88	1.97	2.798 (7)	155
N2—H2n2⋯O4	0.88	1.94	2.798 (7)	165
N2—H2n1⋯O8	0.88	1.94	2.759 (6)	155

Symmetry code: (i) 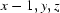.

**Fig. 1.**
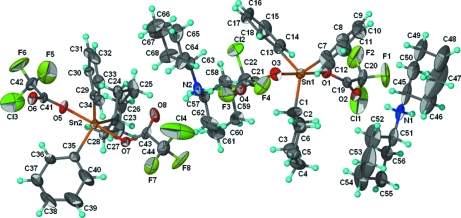
50% Probability displacement plot of the two independent formula units of [(C6H11)2NH2)][Sn(C6H11)(C6H5)2(O2CClF2)2] (I). Hydrogen atoms are drawn as spheres of arbitrary radius.

**Fig. 2.**
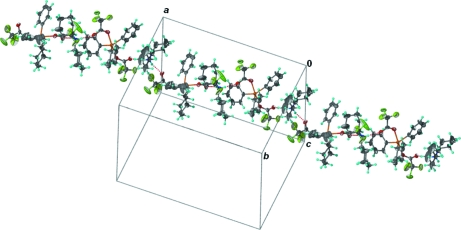
Supramolecular chain formation in (I) with hydrogen bonding interactions shown as dotted lines.

## supplementary crystallographic information

### Comment

Mixed alkyl/diaryltin compounds possess much more useful activity against plant pathogens than the symmetrical triorganotin homologs, particularly if one of the alkyl substituent is a cyclic unit. An earlier study reported the bis(chlorodifluroacetato)cyclohexyldiphenylstannate ion as its bis(1,2-diphenylphosphino)ethane-chelated silver salt (Teo *et al.*, 2007).

Replacing the cation by the dicyclohexylammonium cation furnishes a similar stannate (I, Fig. 1 & Table 1). However, the cation engages in hydrogen bonding to give rise to a chain structure (Fig. 2 & Table 2). The dicyclohexylammonium counterion is particularly effective in stabilizing di(carboxylato)triorganostannates, as shown by previous studies (Ng & Hook, 1999; Ng & Kumar Das, 1992; 1993; Ng & Rae, 2000; Ng *et al.*, 1990; 1991*a*, b,c; 1992; 2000).

### Experimental

Cyclohexyldiphenyltin hydroxide (Teo *et al.*, 2007) (0.38 g, 1 mmol) was added to an ethanol solution (50 ml) of dicyclohexylamine (0.20 ml, 2 mmol) and chlorodifluoroacetic acid (0.1 ml, 2 mmol). The solution was heated to dissolve the reactants completely; the filtered solution yielded the salt in 70% yield.

### Refinement

Carbon-bound H-atoms were placed in calculated positions (C—H 0.95 to 1.00 Å) and were included in the refinement in the riding model approximation, with *U*(H) set to 1.2*U*_eq_(C). The ammonium H atoms (N–H 0.88 Å) were similarly treated.

For the cyclohexylammonium cation, C–C distances were restrained to 1.50±0.01 Å; the anisotropic displacement factors of the C atoms were restrained to be nearly isotropic. Those of the Cl and F atoms were similarly restrained.

There is minor disorder in the chlorodifluoromethyl groups; attempts to treat the group as two overlapping groups required numerous restraints but they did not lead to more meaningful outcome. The final difference Fourier map had a large peak at 1 Å from Sn1. The deepest hole is 1 Å from Cl4.

### Crystal data

**Table:**

(C_12_H_24_N)[Sn(C_6_H_5_)_2_(C_6_H_11_)(CClF_2_O_2_)_2_]	*F*_000_ = 3256
*M**_r_* = 796.29	*D*_x_ = 1.452 Mg m^−^^3^
Monoclinic, *P*2_1_/*c*	Mo *K*α radiation λ = 0.71073 Å
Hall symbol: -P 2ybc	Cell parameters from 9887 reflections
*a* = 22.5469 (3) Å	θ = 2.4–26.7º
*b* = 17.2799 (2) Å	µ = 0.91 mm^−^^1^
*c* = 18.6963 (2) Å	*T* = 100 (2) K
β = 90.324 (1)º	Block, colorless
*V* = 7284 (2) Å^3^	0.15 × 0.15 × 0.10 mm
*Z* = 8	

### Data collection

**Table:**

Bruker SMART APEXII diffractometer	16710 independent reflections
Radiation source: fine-focus sealed tube	12483 reflections with *I* > 2σ(*I*)
Monochromator: graphite	*R*_int_ = 0.033
*T* = 100(2) K	θ_max_ = 27.5º
ω scans	θ_min_ = 1.5º
Absorption correction: multi-scan(SADABS; Sheldrick, 1996)	*h* = −23→29
*T*_min_ = 0.788, *T*_max_ = 0.915	*k* = −22→22
93218 measured reflections	*l* = −24→24

### Refinement

**Table:**

Refinement on *F*^2^	Secondary atom site location: difference Fourier map
Least-squares matrix: full	Hydrogen site location: inferred from neighbouring sites
*R*[*F*^2^ > 2σ(*F*^2^)] = 0.066	H-atom parameters constrained
*wR*(*F*^2^) = 0.216	*w* = 1/[σ^2^(*F*_o_^2^) + (0.1*P*)^2^ + 10.*P*] where *P* = (*F*_o_^2^ + 2*F*_c_^2^)/3
*S* = 1.33	(Δ/σ)_max_ = 0.001
16710 reflections	Δρ_max_ = 2.24 e Å^−^^3^
829 parameters	Δρ_min_ = −2.03 e Å^−^^3^
240 restraints	Extinction correction: none
Primary atom site location: structure-invariant direct methods	

### Fractional atomic coordinates and isotropic or equivalent isotropic displacement parameters (Å^2^)

**Table:**

	*x*	*y*	*z*	*U*_iso_*/*U*_eq_	
Sn1	0.284701 (16)	0.222219 (19)	0.298255 (19)	0.03342 (12)	
Sn2	0.775686 (16)	0.22690 (2)	0.496405 (19)	0.03377 (12)	
Cl1	0.13872 (17)	0.4284 (2)	0.4183 (2)	0.1571 (17)	
Cl2	0.44626 (10)	0.03716 (11)	0.19078 (10)	0.0721 (5)	
Cl3	0.9786 (2)	0.3980 (2)	0.49323 (19)	0.1445 (15)	
Cl4	0.5730 (3)	0.0456 (3)	0.4528 (2)	0.214 (3)	
F1	0.0785 (2)	0.3704 (3)	0.3231 (4)	0.1029 (18)	
F2	0.1537 (3)	0.4350 (4)	0.2914 (4)	0.132 (3)	
F3	0.4614 (3)	0.0058 (3)	0.3216 (3)	0.0965 (17)	
F4	0.3759 (2)	−0.0238 (2)	0.2785 (3)	0.0901 (16)	
F5	0.8999 (4)	0.4183 (4)	0.3985 (5)	0.165 (3)	
F6	0.9681 (3)	0.3555 (5)	0.3664 (3)	0.127 (2)	
F7	0.6311 (2)	0.0371 (3)	0.5612 (3)	0.0911 (16)	
F8	0.5625 (2)	0.1215 (3)	0.5607 (3)	0.110 (2)	
O1	0.22169 (17)	0.3198 (2)	0.3140 (2)	0.0401 (9)	
O2	0.14688 (19)	0.2487 (3)	0.3569 (2)	0.0493 (10)	
O3	0.3450 (2)	0.1222 (2)	0.2786 (2)	0.0497 (10)	
O4	0.4272 (2)	0.1529 (3)	0.3378 (3)	0.0597 (12)	
O5	0.85359 (17)	0.3027 (2)	0.4714 (2)	0.0467 (10)	
O6	0.9297 (2)	0.2306 (3)	0.4326 (3)	0.0521 (11)	
O7	0.69738 (17)	0.1509 (2)	0.5241 (2)	0.0414 (9)	
O8	0.6311 (2)	0.2002 (3)	0.4491 (3)	0.0582 (12)	
N1	0.0384 (2)	0.1755 (3)	0.3823 (3)	0.0535 (14)	
H1N1	0.0693	0.2056	0.3755	0.064*	
H1N2	0.0080	0.2056	0.3921	0.064*	
N2	0.5314 (2)	0.2345 (3)	0.3689 (3)	0.0471 (12)	
H2N1	0.5581	0.2098	0.3948	0.056*	
H2N2	0.4998	0.2046	0.3666	0.056*	
C1	0.2933 (4)	0.2025 (4)	0.4110 (3)	0.0546 (17)	
H1	0.3374	0.1993	0.4153	0.066*	
C2	0.2818 (4)	0.2676 (4)	0.4589 (4)	0.0542 (16)	
H2A	0.2395	0.2822	0.4545	0.065*	
H2B	0.3057	0.3124	0.4428	0.065*	
C3	0.2955 (4)	0.2525 (5)	0.5361 (4)	0.072 (2)	
H3A	0.3389	0.2570	0.5436	0.086*	
H3B	0.2763	0.2930	0.5654	0.086*	
C4	0.2763 (4)	0.1775 (5)	0.5611 (4)	0.075 (2)	
H4A	0.2330	0.1796	0.5691	0.091*	
H4B	0.2955	0.1673	0.6079	0.091*	
C5	0.2888 (4)	0.1125 (4)	0.5141 (4)	0.0620 (18)	
H5	0.3032	0.0645	0.5317	0.074*	
C6	0.2774 (3)	0.1261 (4)	0.4361 (4)	0.0527 (15)	
H6A	0.3000	0.0873	0.4083	0.063*	
H6B	0.2347	0.1174	0.4262	0.063*	
C7	0.2255 (3)	0.1671 (3)	0.2256 (3)	0.0472 (14)	
C8	0.1881 (3)	0.2108 (4)	0.1814 (3)	0.0529 (16)	
H8	0.1871	0.2655	0.1862	0.063*	
C9	0.1523 (4)	0.1746 (5)	0.1306 (4)	0.067 (2)	
H9	0.1288	0.2051	0.0991	0.081*	
C10	0.1504 (4)	0.0959 (6)	0.1256 (4)	0.076 (2)	
H10	0.1238	0.0718	0.0929	0.091*	
C11	0.1864 (4)	0.0523 (5)	0.1670 (5)	0.084 (3)	
H11	0.1874	−0.0022	0.1605	0.100*	
C12	0.2225 (4)	0.0868 (4)	0.2198 (5)	0.074 (2)	
H12	0.2449	0.0552	0.2515	0.089*	
C13	0.3433 (2)	0.3020 (3)	0.2476 (3)	0.0400 (12)	
C14	0.3381 (3)	0.3809 (4)	0.2582 (4)	0.0538 (16)	
H14	0.3086	0.4007	0.2893	0.065*	
C15	0.3768 (4)	0.4319 (4)	0.2224 (5)	0.075 (2)	
H15	0.3738	0.4862	0.2293	0.090*	
C16	0.4199 (4)	0.4013 (6)	0.1767 (5)	0.082 (3)	
H16	0.4463	0.4352	0.1527	0.098*	
C17	0.4244 (3)	0.3234 (5)	0.1660 (4)	0.066 (2)	
H17	0.4536	0.3032	0.1347	0.079*	
C18	0.3867 (3)	0.2750 (4)	0.2007 (3)	0.0505 (15)	
H18	0.3900	0.2209	0.1928	0.061*	
C19	0.1706 (3)	0.3086 (3)	0.3373 (3)	0.0381 (12)	
C20	0.1339 (3)	0.3826 (4)	0.3392 (5)	0.063 (2)	
C21	0.3960 (3)	0.1105 (3)	0.3025 (3)	0.0440 (13)	
C22	0.4191 (3)	0.0316 (4)	0.2792 (4)	0.0586 (17)	
C23	0.7271 (2)	0.3332 (3)	0.5050 (3)	0.0410 (13)	
H23	0.6854	0.3196	0.5181	0.049*	
C24	0.7247 (3)	0.3753 (3)	0.4336 (4)	0.0488 (15)	
H24A	0.7064	0.3413	0.3971	0.059*	
H24B	0.7655	0.3876	0.4180	0.059*	
C25	0.6888 (3)	0.4500 (4)	0.4394 (5)	0.069 (2)	
H25A	0.6899	0.4781	0.3933	0.083*	
H25B	0.6470	0.4375	0.4502	0.083*	
C26	0.7143 (3)	0.5010 (4)	0.4983 (6)	0.078 (3)	
H26A	0.7548	0.5172	0.4850	0.093*	
H26B	0.6896	0.5481	0.5027	0.093*	
C27	0.7164 (3)	0.4598 (5)	0.5694 (5)	0.074 (2)	
H27A	0.7350	0.4939	0.6057	0.089*	
H27B	0.6755	0.4480	0.5852	0.089*	
C28	0.7514 (3)	0.3856 (4)	0.5634 (4)	0.0529 (16)	
H28A	0.7503	0.3579	0.6097	0.063*	
H28B	0.7933	0.3981	0.5530	0.063*	
C29	0.7813 (2)	0.1707 (3)	0.3953 (3)	0.0345 (11)	
C30	0.7970 (3)	0.2110 (3)	0.3339 (3)	0.0407 (12)	
H30	0.8062	0.2645	0.3371	0.049*	
C31	0.7994 (3)	0.1744 (4)	0.2682 (3)	0.0517 (15)	
H31	0.8103	0.2027	0.2268	0.062*	
C32	0.7860 (3)	0.0963 (4)	0.2628 (4)	0.0595 (19)	
H32	0.7870	0.0713	0.2175	0.071*	
C33	0.7714 (3)	0.0555 (4)	0.3226 (4)	0.0556 (17)	
H33	0.7635	0.0016	0.3192	0.067*	
C34	0.7681 (3)	0.0926 (3)	0.3886 (3)	0.0449 (13)	
H34	0.7566	0.0640	0.4297	0.054*	
C35	0.8191 (3)	0.1774 (4)	0.5875 (3)	0.0477 (14)	
C36	0.8771 (4)	0.1882 (8)	0.6018 (4)	0.100 (4)	
H36	0.8997	0.2185	0.5696	0.120*	
C37	0.9055 (5)	0.1574 (10)	0.6611 (5)	0.122 (5)	
H37	0.9465	0.1668	0.6690	0.147*	
C38	0.8746 (5)	0.1139 (6)	0.7075 (5)	0.099 (4)	
H38	0.8947	0.0863	0.7444	0.119*	
C39	0.8136 (6)	0.1093 (7)	0.7016 (6)	0.127 (5)	
H39	0.7903	0.0874	0.7387	0.152*	
C40	0.7869 (5)	0.1383 (7)	0.6382 (6)	0.121 (5)	
H40	0.7456	0.1306	0.6305	0.145*	
C41	0.9035 (2)	0.2914 (4)	0.4443 (3)	0.0406 (12)	
C42	0.9356 (3)	0.3665 (5)	0.4250 (4)	0.066 (2)	
C43	0.6485 (3)	0.1538 (3)	0.4938 (3)	0.0415 (13)	
C44	0.6046 (3)	0.0908 (4)	0.5189 (4)	0.0644 (19)	
C45	0.0254 (3)	0.1347 (5)	0.3142 (5)	0.078 (3)	
H45	0.0609	0.1037	0.3000	0.093*	
C46	−0.0273 (5)	0.0812 (7)	0.3223 (6)	0.123 (4)	
H46A	−0.0633	0.1123	0.3323	0.148*	
H46B	−0.0205	0.0462	0.3634	0.148*	
C47	−0.0372 (8)	0.0343 (8)	0.2561 (7)	0.176 (6)	
H47A	−0.0011	0.0049	0.2435	0.211*	
H47B	−0.0704	−0.0024	0.2625	0.211*	
C48	−0.0515 (5)	0.0922 (7)	0.2003 (6)	0.107 (4)	
H48	−0.0879	0.0922	0.1744	0.128*	
C49	−0.0025 (5)	0.1524 (7)	0.1877 (5)	0.117 (4)	
H49A	−0.0160	0.1908	0.1519	0.140*	
H49B	0.0334	0.1265	0.1690	0.140*	
C50	0.0124 (4)	0.1931 (6)	0.2575 (4)	0.095 (3)	
H50A	0.0473	0.2270	0.2508	0.114*	
H50B	−0.0215	0.2258	0.2723	0.114*	
C51	0.0505 (3)	0.1262 (3)	0.4461 (4)	0.0616 (19)	
H51	0.0138	0.0960	0.4565	0.074*	
C52	0.1002 (5)	0.0695 (6)	0.4357 (6)	0.114 (4)	
H52A	0.0900	0.0336	0.3962	0.136*	
H52B	0.1368	0.0976	0.4226	0.136*	
C53	0.1106 (6)	0.0242 (7)	0.5036 (7)	0.132 (4)	
H53A	0.1447	−0.0108	0.4961	0.158*	
H53B	0.0753	−0.0084	0.5125	0.158*	
C54	0.1224 (5)	0.0714 (7)	0.5684 (7)	0.131 (5)	
H54A	0.1606	0.0991	0.5635	0.157*	
H54B	0.1248	0.0375	0.6110	0.157*	
C55	0.0729 (4)	0.1283 (5)	0.5769 (5)	0.090 (3)	
H55A	0.0820	0.1633	0.6174	0.108*	
H55B	0.0359	0.1002	0.5882	0.108*	
C56	0.0636 (3)	0.1759 (4)	0.5097 (4)	0.0640 (19)	
H56A	0.0302	0.2122	0.5171	0.077*	
H56B	0.0996	0.2069	0.5004	0.077*	
C57	0.5143 (3)	0.3081 (4)	0.4094 (3)	0.0517 (15)	
H57	0.5502	0.3417	0.4133	0.062*	
C58	0.4662 (3)	0.3537 (4)	0.3721 (3)	0.0557 (16)	
H58A	0.4309	0.3204	0.3644	0.067*	
H58B	0.4804	0.3714	0.3249	0.067*	
C59	0.4495 (3)	0.4228 (4)	0.4175 (3)	0.0589 (17)	
H59A	0.4836	0.4589	0.4198	0.071*	
H59B	0.4160	0.4503	0.3944	0.071*	
C60	0.4321 (3)	0.4006 (4)	0.4925 (3)	0.0566 (17)	
H60A	0.4240	0.4480	0.5206	0.068*	
H60B	0.3953	0.3694	0.4909	0.068*	
C61	0.4800 (4)	0.3552 (5)	0.5284 (4)	0.082 (3)	
H61A	0.4666	0.3386	0.5763	0.098*	
H61B	0.5155	0.3882	0.5347	0.098*	
C62	0.4961 (5)	0.2842 (5)	0.4841 (4)	0.083 (3)	
H62A	0.5292	0.2561	0.5076	0.100*	
H62B	0.4616	0.2489	0.4813	0.100*	
C63	0.5551 (3)	0.2424 (4)	0.2958 (4)	0.0548 (16)	
H63	0.5229	0.2638	0.2646	0.066*	
C64	0.5719 (4)	0.1637 (4)	0.2675 (4)	0.075 (2)	
H64A	0.5376	0.1283	0.2712	0.090*	
H64B	0.6047	0.1421	0.2969	0.090*	
C65	0.5914 (4)	0.1684 (6)	0.1899 (4)	0.088 (3)	
H65A	0.6019	0.1163	0.1722	0.105*	
H65B	0.5586	0.1888	0.1599	0.105*	
C66	0.6437 (4)	0.2207 (4)	0.1852 (5)	0.082 (3)	
H66A	0.6549	0.2268	0.1344	0.099*	
H66B	0.6776	0.1966	0.2106	0.099*	
C67	0.6317 (6)	0.2998 (5)	0.2171 (5)	0.106 (3)	
H67A	0.6691	0.3299	0.2180	0.128*	
H67B	0.6031	0.3277	0.1861	0.128*	
C68	0.6073 (4)	0.2957 (5)	0.2917 (4)	0.078 (2)	
H68A	0.6387	0.2773	0.3248	0.093*	
H68B	0.5952	0.3481	0.3071	0.093*	

### Atomic displacement parameters (Å^2^)

**Table:**

	*U*^11^	*U*^22^	*U*^33^	*U*^12^	*U*^13^	*U*^23^
Sn1	0.0336 (2)	0.02637 (19)	0.0404 (2)	−0.00191 (13)	0.00458 (15)	−0.00010 (13)
Sn2	0.0254 (2)	0.0388 (2)	0.0371 (2)	0.00561 (13)	0.00435 (14)	0.00539 (14)
Cl1	0.120 (3)	0.137 (3)	0.214 (4)	0.010 (2)	0.019 (2)	−0.125 (3)
Cl2	0.0926 (15)	0.0621 (10)	0.0618 (10)	0.0017 (10)	0.0293 (10)	−0.0192 (8)
Cl3	0.196 (4)	0.114 (2)	0.124 (2)	−0.087 (2)	−0.012 (2)	−0.0278 (19)
Cl4	0.353 (6)	0.173 (3)	0.114 (2)	−0.182 (4)	−0.079 (3)	0.026 (2)
F1	0.037 (2)	0.085 (3)	0.187 (5)	0.011 (2)	−0.017 (3)	−0.013 (3)
F2	0.098 (4)	0.092 (4)	0.206 (6)	0.040 (3)	0.046 (4)	0.073 (4)
F3	0.094 (4)	0.103 (4)	0.093 (3)	0.061 (3)	0.010 (3)	0.027 (3)
F4	0.093 (4)	0.041 (2)	0.137 (4)	−0.001 (2)	0.036 (3)	0.014 (2)
F5	0.165 (6)	0.114 (5)	0.214 (7)	0.028 (4)	−0.001 (5)	0.105 (5)
F6	0.128 (5)	0.169 (5)	0.087 (3)	−0.056 (4)	0.055 (3)	0.013 (4)
F7	0.077 (3)	0.059 (3)	0.137 (4)	0.008 (2)	0.021 (3)	0.050 (3)
F8	0.087 (3)	0.099 (4)	0.146 (4)	0.038 (3)	0.083 (3)	0.060 (3)
O1	0.035 (2)	0.0314 (18)	0.054 (2)	0.0011 (15)	0.0059 (17)	0.0039 (16)
O2	0.040 (2)	0.046 (2)	0.062 (3)	−0.0093 (19)	0.007 (2)	−0.004 (2)
O3	0.051 (3)	0.038 (2)	0.060 (3)	0.0083 (19)	−0.004 (2)	−0.0127 (18)
O4	0.046 (3)	0.080 (3)	0.054 (3)	0.004 (2)	0.000 (2)	−0.014 (2)
O5	0.028 (2)	0.047 (2)	0.066 (3)	0.0013 (17)	0.0140 (18)	−0.003 (2)
O6	0.036 (2)	0.060 (3)	0.061 (3)	0.0089 (19)	0.009 (2)	−0.005 (2)
O7	0.033 (2)	0.045 (2)	0.046 (2)	0.0036 (17)	0.0068 (17)	0.0152 (17)
O8	0.038 (2)	0.043 (2)	0.094 (4)	−0.0035 (19)	−0.018 (2)	0.022 (2)
N1	0.029 (3)	0.045 (3)	0.086 (4)	−0.008 (2)	0.011 (3)	−0.025 (3)
N2	0.030 (3)	0.057 (3)	0.054 (3)	0.017 (2)	−0.006 (2)	−0.014 (2)
C1	0.080 (5)	0.039 (3)	0.044 (3)	0.003 (3)	0.010 (3)	0.006 (3)
C2	0.061 (4)	0.051 (4)	0.050 (4)	−0.005 (3)	−0.002 (3)	−0.001 (3)
C3	0.088 (6)	0.075 (5)	0.053 (4)	−0.017 (5)	0.004 (4)	0.003 (4)
C4	0.092 (6)	0.083 (6)	0.051 (4)	−0.017 (5)	−0.014 (4)	0.012 (4)
C5	0.071 (5)	0.056 (4)	0.059 (4)	0.001 (4)	0.004 (4)	0.022 (3)
C6	0.061 (4)	0.040 (3)	0.057 (4)	−0.004 (3)	−0.001 (3)	0.010 (3)
C7	0.047 (4)	0.038 (3)	0.056 (3)	−0.010 (3)	−0.002 (3)	0.003 (3)
C8	0.061 (4)	0.055 (4)	0.043 (3)	−0.001 (3)	0.004 (3)	−0.003 (3)
C9	0.057 (4)	0.084 (6)	0.062 (4)	−0.013 (4)	−0.004 (3)	0.014 (4)
C10	0.076 (6)	0.091 (6)	0.060 (4)	−0.016 (5)	−0.010 (4)	−0.019 (4)
C11	0.093 (7)	0.062 (5)	0.096 (6)	−0.031 (5)	−0.013 (5)	−0.016 (4)
C12	0.095 (6)	0.040 (4)	0.086 (5)	−0.009 (4)	−0.036 (5)	−0.006 (3)
C13	0.030 (3)	0.047 (3)	0.042 (3)	−0.010 (2)	−0.005 (2)	0.006 (2)
C14	0.046 (4)	0.045 (3)	0.071 (4)	−0.018 (3)	−0.007 (3)	0.010 (3)
C15	0.063 (5)	0.046 (4)	0.114 (7)	−0.025 (4)	−0.020 (5)	0.024 (4)
C16	0.040 (4)	0.114 (8)	0.091 (6)	−0.025 (5)	0.005 (4)	0.035 (5)
C17	0.046 (4)	0.091 (6)	0.061 (4)	−0.007 (4)	0.006 (3)	0.017 (4)
C18	0.036 (3)	0.071 (4)	0.044 (3)	−0.006 (3)	0.002 (3)	0.004 (3)
C19	0.035 (3)	0.037 (3)	0.043 (3)	−0.004 (2)	−0.004 (2)	−0.002 (2)
C20	0.031 (3)	0.048 (4)	0.110 (6)	0.004 (3)	0.015 (4)	0.004 (4)
C21	0.051 (4)	0.043 (3)	0.038 (3)	0.009 (3)	0.009 (3)	−0.002 (2)
C22	0.061 (4)	0.049 (4)	0.066 (4)	0.016 (3)	0.011 (3)	0.005 (3)
C23	0.024 (3)	0.038 (3)	0.061 (4)	0.001 (2)	0.010 (2)	−0.006 (2)
C24	0.038 (3)	0.036 (3)	0.072 (4)	0.008 (2)	−0.006 (3)	−0.002 (3)
C25	0.046 (4)	0.033 (3)	0.128 (7)	0.012 (3)	−0.017 (4)	−0.002 (4)
C26	0.041 (4)	0.038 (3)	0.154 (9)	0.009 (3)	−0.001 (5)	−0.023 (4)
C27	0.049 (4)	0.066 (5)	0.107 (7)	−0.001 (4)	0.019 (4)	−0.038 (5)
C28	0.039 (3)	0.057 (4)	0.063 (4)	−0.002 (3)	0.011 (3)	−0.016 (3)
C29	0.029 (3)	0.034 (2)	0.041 (3)	0.010 (2)	0.005 (2)	0.007 (2)
C30	0.037 (3)	0.045 (3)	0.040 (3)	0.007 (2)	0.005 (2)	0.010 (2)
C31	0.051 (4)	0.064 (4)	0.040 (3)	0.013 (3)	0.000 (3)	0.006 (3)
C32	0.062 (4)	0.072 (5)	0.045 (3)	0.031 (4)	−0.013 (3)	−0.009 (3)
C33	0.057 (4)	0.040 (3)	0.070 (4)	0.012 (3)	−0.011 (3)	−0.007 (3)
C34	0.046 (3)	0.036 (3)	0.052 (3)	0.010 (2)	0.000 (3)	0.006 (2)
C35	0.044 (3)	0.061 (4)	0.038 (3)	0.016 (3)	−0.003 (2)	0.000 (3)
C36	0.049 (5)	0.212 (12)	0.039 (4)	−0.003 (6)	−0.009 (3)	0.029 (6)
C37	0.057 (6)	0.260 (17)	0.050 (5)	0.027 (8)	−0.013 (4)	0.008 (7)
C38	0.109 (8)	0.114 (8)	0.075 (6)	0.036 (7)	−0.051 (6)	0.002 (5)
C39	0.122 (10)	0.144 (10)	0.114 (9)	−0.011 (8)	−0.047 (7)	0.084 (8)
C40	0.094 (8)	0.143 (10)	0.125 (9)	−0.033 (7)	−0.054 (7)	0.093 (8)
C41	0.027 (3)	0.051 (3)	0.044 (3)	0.002 (2)	0.003 (2)	0.008 (2)
C42	0.043 (4)	0.068 (5)	0.086 (5)	0.007 (3)	0.020 (4)	0.032 (4)
C43	0.031 (3)	0.033 (3)	0.060 (3)	0.000 (2)	0.011 (3)	0.004 (2)
C44	0.056 (4)	0.051 (4)	0.086 (5)	−0.013 (3)	0.004 (4)	0.009 (4)
C45	0.043 (4)	0.084 (5)	0.106 (6)	−0.011 (4)	0.012 (4)	−0.057 (5)
C46	0.105 (7)	0.127 (7)	0.137 (7)	−0.061 (6)	0.009 (6)	−0.066 (6)
C47	0.172 (10)	0.166 (9)	0.189 (10)	−0.078 (8)	−0.017 (8)	−0.065 (8)
C48	0.073 (5)	0.140 (7)	0.108 (6)	−0.040 (5)	−0.016 (5)	−0.056 (6)
C49	0.089 (6)	0.176 (8)	0.086 (6)	−0.026 (6)	0.010 (5)	−0.069 (6)
C50	0.064 (5)	0.148 (7)	0.073 (5)	−0.018 (5)	0.005 (4)	−0.058 (5)
C51	0.039 (3)	0.036 (3)	0.110 (5)	0.002 (3)	0.009 (3)	0.007 (3)
C52	0.096 (7)	0.084 (6)	0.161 (8)	0.044 (5)	0.010 (6)	−0.007 (6)
C53	0.108 (7)	0.100 (7)	0.187 (9)	0.051 (6)	0.015 (7)	0.054 (7)
C54	0.107 (7)	0.124 (8)	0.162 (9)	0.035 (6)	−0.008 (7)	0.060 (7)
C55	0.061 (5)	0.093 (6)	0.116 (6)	−0.001 (4)	−0.007 (4)	0.057 (5)
C56	0.053 (4)	0.051 (4)	0.089 (5)	−0.003 (3)	−0.007 (4)	0.021 (3)
C57	0.045 (3)	0.053 (3)	0.057 (3)	0.017 (3)	0.001 (3)	−0.014 (3)
C58	0.057 (4)	0.057 (4)	0.053 (3)	0.018 (3)	−0.002 (3)	−0.005 (3)
C59	0.062 (4)	0.052 (4)	0.064 (4)	0.017 (3)	0.008 (3)	−0.001 (3)
C60	0.063 (4)	0.045 (3)	0.061 (4)	0.012 (3)	0.013 (3)	−0.013 (3)
C61	0.102 (6)	0.081 (5)	0.062 (4)	0.036 (5)	0.002 (4)	−0.017 (4)
C62	0.108 (6)	0.080 (5)	0.062 (4)	0.056 (5)	−0.005 (4)	−0.014 (4)
C63	0.048 (4)	0.059 (4)	0.057 (4)	0.019 (3)	−0.001 (3)	−0.010 (3)
C64	0.069 (5)	0.069 (4)	0.087 (5)	−0.004 (4)	0.025 (4)	−0.031 (4)
C65	0.076 (5)	0.100 (6)	0.088 (5)	0.018 (5)	0.005 (4)	−0.040 (5)
C66	0.097 (6)	0.075 (5)	0.076 (5)	0.022 (4)	0.033 (5)	−0.003 (4)
C67	0.137 (8)	0.078 (5)	0.106 (6)	0.011 (6)	0.043 (6)	0.002 (5)
C68	0.104 (6)	0.054 (4)	0.076 (5)	0.010 (4)	0.025 (4)	−0.009 (4)

### Geometric parameters (Å, °)

**Table:**

Sn1—C1	2.144 (6)	C28—H28A	0.9900
Sn1—C7	2.125 (6)	C28—H28B	0.9900
Sn1—C13	2.134 (6)	C29—C34	1.388 (8)
Sn1—O1	2.226 (4)	C29—C30	1.389 (7)
Sn1—O3	2.231 (4)	C30—C31	1.382 (9)
Sn2—C23	2.145 (5)	C30—H30	0.9500
Sn2—C29	2.130 (5)	C31—C32	1.386 (10)
Sn2—C35	2.138 (6)	C31—H31	0.9500
Sn2—O5	2.243 (4)	C32—C33	1.363 (10)
Sn2—O7	2.263 (4)	C32—H32	0.9500
Cl1—C20	1.682 (9)	C33—C34	1.394 (9)
Cl2—C22	1.769 (7)	C33—H33	0.9500
Cl3—C42	1.689 (10)	C34—H34	0.9500
Cl4—C44	1.623 (9)	C35—C36	1.347 (10)
F1—C20	1.299 (8)	C35—C40	1.374 (12)
F2—C20	1.350 (9)	C36—C37	1.383 (12)
F3—C22	1.315 (9)	C36—H36	0.9500
F4—C22	1.365 (9)	C37—C38	1.345 (16)
F5—C42	1.298 (9)	C37—H37	0.9500
F6—C42	1.336 (9)	C38—C39	1.382 (16)
F7—C44	1.355 (9)	C38—H38	0.9500
F8—C44	1.341 (9)	C39—C40	1.420 (12)
O1—C19	1.248 (7)	C39—H39	0.9500
O2—C19	1.223 (7)	C40—H40	0.9500
O3—C21	1.247 (8)	C41—C42	1.531 (9)
O4—C21	1.209 (8)	C43—C44	1.546 (9)
O5—C41	1.251 (7)	C45—C50	1.491 (9)
O6—C41	1.224 (7)	C45—C46	1.514 (8)
O7—C43	1.236 (7)	C45—H45	1.0000
O8—C43	1.222 (7)	C46—C47	1.495 (9)
N1—C51	1.490 (9)	C46—H46A	0.9900
N1—C45	1.482 (9)	C46—H46B	0.9900
N1—H1N1	0.8800	C47—C48	1.479 (10)
N1—H1N2	0.8800	C47—H47A	0.9900
N2—C63	1.476 (9)	C47—H47B	0.9900
N2—C57	1.531 (8)	C48—C49	1.538 (9)
N2—H2N1	0.8800	C48—H48	0.9500
N2—H2N2	0.8800	C49—C50	1.517 (8)
C1—C6	1.447 (8)	C49—H49A	0.9900
C1—C2	1.461 (9)	C49—H49B	0.9900
C1—H1	1.0000	C50—H50A	0.9900
C2—C3	1.499 (10)	C50—H50B	0.9900
C2—H2A	0.9900	C51—C56	1.494 (8)
C2—H2B	0.9900	C51—C52	1.501 (8)
C3—C4	1.446 (11)	C51—H51	1.0000
C3—H3A	0.9900	C52—C53	1.508 (9)
C3—H3B	0.9900	C52—H52A	0.9900
C4—C5	1.454 (11)	C52—H52B	0.9900
C4—H4A	0.9900	C53—C54	1.483 (9)
C4—H4B	0.9900	C53—H53A	0.9900
C5—C6	1.497 (9)	C53—H53B	0.9900
C5—H5	0.9500	C54—C55	1.496 (9)
C6—H6A	0.9900	C54—H54A	0.9900
C6—H6B	0.9900	C54—H54B	0.9900
C7—C12	1.394 (9)	C55—C56	1.515 (8)
C7—C8	1.398 (9)	C55—H55A	0.9900
C8—C9	1.392 (10)	C55—H55B	0.9900
C8—H8	0.9500	C56—H56A	0.9900
C9—C10	1.364 (12)	C56—H56B	0.9900
C9—H9	0.9500	C57—C58	1.507 (7)
C10—C11	1.348 (13)	C57—C62	1.516 (8)
C10—H10	0.9500	C57—H57	1.0000
C11—C12	1.407 (11)	C58—C59	1.513 (7)
C11—H11	0.9500	C58—H58A	0.9900
C12—H12	0.9500	C58—H58B	0.9900
C13—C14	1.382 (9)	C59—C60	1.509 (7)
C13—C18	1.398 (9)	C59—H59A	0.9900
C14—C15	1.411 (9)	C59—H59B	0.9900
C14—H14	0.9500	C60—C61	1.492 (8)
C15—C16	1.401 (13)	C60—H60A	0.9900
C15—H15	0.9500	C60—H60B	0.9900
C16—C17	1.364 (13)	C61—C62	1.524 (7)
C16—H16	0.9500	C61—H61A	0.9900
C17—C18	1.360 (10)	C61—H61B	0.9900
C17—H17	0.9500	C62—H62A	0.9900
C18—H18	0.9500	C62—H62B	0.9900
C19—C20	1.524 (9)	C63—C68	1.497 (8)
C21—C22	1.524 (9)	C63—C64	1.510 (7)
C23—C28	1.520 (8)	C63—H63	1.0000
C23—C24	1.521 (9)	C64—C65	1.520 (8)
C23—H23	1.0000	C64—H64A	0.9900
C24—C25	1.527 (8)	C64—H64B	0.9900
C24—H24A	0.9900	C65—C66	1.488 (9)
C24—H24B	0.9900	C65—H65A	0.9900
C25—C26	1.518 (11)	C65—H65B	0.9900
C25—H25A	0.9900	C66—C67	1.517 (8)
C25—H25B	0.9900	C66—H66A	0.9900
C26—C27	1.508 (13)	C66—H66B	0.9900
C26—H26A	0.9900	C67—C68	1.503 (8)
C26—H26B	0.9900	C67—H67A	0.9900
C27—C28	1.511 (10)	C67—H67B	0.9900
C27—H27A	0.9900	C68—H68A	0.9900
C27—H27B	0.9900	C68—H68B	0.9900
			
C7—Sn1—C13	113.2 (2)	C36—C35—C40	116.6 (7)
C7—Sn1—C1	127.6 (3)	C36—C35—Sn2	122.9 (6)
C13—Sn1—C1	119.1 (3)	C40—C35—Sn2	120.3 (5)
C7—Sn1—O1	91.4 (2)	C35—C36—C37	123.4 (10)
C13—Sn1—O1	88.04 (19)	C35—C36—H36	118.3
C1—Sn1—O1	92.6 (2)	C37—C36—H36	118.3
C7—Sn1—O3	85.9 (2)	C38—C37—C36	119.6 (10)
C13—Sn1—O3	92.7 (2)	C38—C37—H37	120.2
C1—Sn1—O3	89.3 (2)	C36—C37—H37	120.2
O1—Sn1—O3	177.3 (2)	C37—C38—C39	119.9 (8)
C29—Sn2—C35	119.7 (2)	C37—C38—H38	120.0
C29—Sn2—C23	119.3 (2)	C39—C38—H38	120.0
C35—Sn2—C23	121.0 (2)	C38—C39—C40	117.7 (11)
C29—Sn2—O5	91.75 (18)	C38—C39—H39	121.2
C35—Sn2—O5	92.6 (2)	C40—C39—H39	121.2
C23—Sn2—O5	85.22 (18)	C35—C40—C39	121.7 (10)
C29—Sn2—O7	89.36 (18)	C35—C40—H40	119.1
C35—Sn2—O7	86.5 (2)	C39—C40—H40	119.1
C23—Sn2—O7	94.60 (17)	O6—C41—O5	129.9 (6)
O5—Sn2—O7	178.8 (2)	O6—C41—C42	117.2 (5)
C19—O1—Sn1	121.2 (3)	O5—C41—C42	112.9 (5)
C21—O3—Sn1	128.9 (4)	F5—C42—F6	97.3 (7)
C41—O5—Sn2	134.4 (4)	F5—C42—C41	112.4 (7)
C43—O7—Sn2	124.4 (3)	F6—C42—C41	109.6 (7)
C51—N1—H1N1	108.1	F5—C42—Cl3	114.7 (8)
C45—N1—H1N1	108.1	F6—C42—Cl3	110.5 (6)
C51—N1—H1N2	108.1	C41—C42—Cl3	111.4 (5)
C45—N1—H1N2	108.1	O8—C43—O7	128.4 (5)
H1N1—N1—H1N2	107.3	O8—C43—C44	117.8 (6)
C45—N1—C51	116.8 (6)	O7—C43—C44	113.7 (5)
C57—N2—C63	118.3 (5)	F8—C44—F7	104.0 (6)
C63—N2—H2N1	107.7	F8—C44—C43	110.8 (6)
C57—N2—H2N1	107.7	F7—C44—C43	112.2 (6)
C63—N2—H2N2	107.7	F8—C44—Cl4	108.9 (7)
C57—N2—H2N2	107.7	F7—C44—Cl4	107.7 (6)
H2N1—N2—H2N2	107.1	C43—C44—Cl4	112.7 (6)
C6—C1—C2	117.4 (6)	N1—C45—C50	109.0 (6)
C6—C1—Sn1	116.3 (5)	N1—C45—C46	110.8 (7)
C2—C1—Sn1	117.7 (4)	C50—C45—C46	109.4 (9)
C6—C1—H1	99.9	N1—C45—H45	109.2
C2—C1—H1	99.9	C50—C45—H45	109.2
Sn1—C1—H1	99.9	C46—C45—H45	109.2
C1—C2—C3	114.8 (6)	C45—C46—C47	111.2 (9)
C1—C2—H2A	108.6	C45—C46—H46A	109.4
C3—C2—H2A	108.6	C47—C46—H46A	109.4
C1—C2—H2B	108.6	C45—C46—H46B	109.4
C3—C2—H2B	108.6	C47—C46—H46B	109.4
H2A—C2—H2B	107.5	H46A—C46—H46B	108.0
C4—C3—C2	114.0 (7)	C48—C47—C46	104.4 (11)
C4—C3—H3A	108.8	C48—C47—H47A	110.9
C2—C3—H3A	108.8	C46—C47—H47A	110.9
C4—C3—H3B	108.8	C48—C47—H47B	110.9
C2—C3—H3B	108.8	C46—C47—H47B	110.9
H3A—C3—H3B	107.6	H47A—C47—H47B	108.9
C3—C4—C5	115.9 (7)	C49—C48—C47	114.3 (10)
C3—C4—H4A	108.3	C49—C48—H48	122.9
C5—C4—H4A	108.3	C47—C48—H48	122.9
C3—C4—H4B	108.3	C48—C49—C50	109.7 (8)
C5—C4—H4B	108.3	C48—C49—H49A	109.7
H4A—C4—H4B	107.4	C50—C49—H49A	109.7
C4—C5—C6	115.8 (6)	C48—C49—H49B	109.7
C4—C5—H5	122.1	C50—C49—H49B	109.7
C6—C5—H5	122.1	H49A—C49—H49B	108.2
C1—C6—C5	114.6 (6)	C45—C50—C49	109.8 (9)
C1—C6—H6A	108.6	C45—C50—H50A	109.7
C5—C6—H6A	108.6	C49—C50—H50A	109.7
C1—C6—H6B	108.6	C45—C50—H50B	109.7
C5—C6—H6B	108.6	C49—C50—H50B	109.7
H6A—C6—H6B	107.6	H50A—C50—H50B	108.2
C12—C7—C8	117.6 (6)	N1—C51—C56	110.1 (5)
C12—C7—Sn1	121.8 (5)	N1—C51—C52	113.7 (7)
C8—C7—Sn1	120.7 (5)	C56—C51—C52	109.6 (7)
C9—C8—C7	120.4 (7)	N1—C51—H51	107.7
C9—C8—H8	119.8	C56—C51—H51	107.7
C7—C8—H8	119.8	C52—C51—H51	107.7
C10—C9—C8	120.8 (8)	C53—C52—C51	110.0 (8)
C10—C9—H9	119.6	C53—C52—H52A	109.7
C8—C9—H9	119.6	C51—C52—H52A	109.7
C11—C10—C9	120.0 (8)	C53—C52—H52B	109.7
C11—C10—H10	120.0	C51—C52—H52B	109.7
C9—C10—H10	120.0	H52A—C52—H52B	108.2
C10—C11—C12	120.6 (8)	C52—C53—C54	115.4 (10)
C10—C11—H11	119.7	C52—C53—H53A	108.4
C12—C11—H11	119.7	C54—C53—H53A	108.4
C7—C12—C11	120.3 (8)	C52—C53—H53B	108.4
C7—C12—H12	119.9	C54—C53—H53B	108.4
C11—C12—H12	119.9	H53A—C53—H53B	107.5
C14—C13—C18	118.6 (6)	C53—C54—C55	108.5 (10)
C14—C13—Sn1	121.4 (5)	C53—C54—H54A	110.0
C18—C13—Sn1	120.0 (5)	C55—C54—H54A	110.0
C13—C14—C15	119.7 (7)	C53—C54—H54B	110.0
C13—C14—H14	120.2	C55—C54—H54B	110.0
C15—C14—H14	120.2	H54A—C54—H54B	108.4
C16—C15—C14	119.1 (8)	C54—C55—C56	111.7 (8)
C16—C15—H15	120.4	C54—C55—H55A	109.3
C14—C15—H15	120.4	C56—C55—H55A	109.3
C17—C16—C15	120.9 (7)	C54—C55—H55B	109.3
C17—C16—H16	119.5	C56—C55—H55B	109.3
C15—C16—H16	119.5	H55A—C55—H55B	107.9
C18—C17—C16	119.3 (8)	C51—C56—C55	111.9 (7)
C18—C17—H17	120.4	C51—C56—H56A	109.2
C16—C17—H17	120.4	C55—C56—H56A	109.2
C17—C18—C13	122.4 (7)	C51—C56—H56B	109.2
C17—C18—H18	118.8	C55—C56—H56B	109.2
C13—C18—H18	118.8	H56A—C56—H56B	107.9
O2—C19—O1	130.0 (6)	C58—C57—C62	111.8 (6)
O2—C19—C20	117.7 (5)	C58—C57—N2	112.9 (5)
O1—C19—C20	112.3 (5)	C62—C57—N2	107.4 (5)
F1—C20—F2	106.1 (7)	C58—C57—H57	108.2
F1—C20—C19	112.3 (6)	C62—C57—H57	108.2
F2—C20—C19	111.4 (6)	N2—C57—H57	108.2
F1—C20—Cl1	109.7 (6)	C57—C58—C59	109.5 (5)
F2—C20—Cl1	104.3 (6)	C57—C58—H58A	109.8
C19—C20—Cl1	112.5 (6)	C59—C58—H58A	109.8
O4—C21—O3	129.0 (6)	C57—C58—H58B	109.8
O4—C21—C22	119.9 (6)	C59—C58—H58B	109.8
O3—C21—C22	111.0 (6)	H58A—C58—H58B	108.2
F3—C22—F4	106.4 (6)	C60—C59—C58	112.8 (5)
F3—C22—C21	112.3 (6)	C60—C59—H59A	109.0
F4—C22—C21	112.7 (6)	C58—C59—H59A	109.0
F3—C22—Cl2	109.1 (5)	C60—C59—H59B	109.0
F4—C22—Cl2	106.3 (5)	C58—C59—H59B	109.0
C21—C22—Cl2	109.8 (5)	H59A—C59—H59B	107.8
C28—C23—C24	110.9 (5)	C61—C60—C59	111.1 (6)
C28—C23—Sn2	112.4 (4)	C61—C60—H60A	109.4
C24—C23—Sn2	111.1 (4)	C59—C60—H60A	109.4
C28—C23—H23	107.4	C61—C60—H60B	109.4
C24—C23—H23	107.4	C59—C60—H60B	109.4
Sn2—C23—H23	107.4	H60A—C60—H60B	108.0
C23—C24—C25	111.0 (6)	C60—C61—C62	110.7 (6)
C23—C24—H24A	109.4	C60—C61—H61A	109.5
C25—C24—H24A	109.4	C62—C61—H61A	109.5
C23—C24—H24B	109.4	C60—C61—H61B	109.5
C25—C24—H24B	109.4	C62—C61—H61B	109.5
H24A—C24—H24B	108.0	H61A—C61—H61B	108.1
C26—C25—C24	110.1 (6)	C57—C62—C61	110.3 (6)
C26—C25—H25A	109.6	C57—C62—H62A	109.6
C24—C25—H25A	109.6	C61—C62—H62A	109.6
C26—C25—H25B	109.6	C57—C62—H62B	109.6
C24—C25—H25B	109.6	C61—C62—H62B	109.6
H25A—C25—H25B	108.1	H62A—C62—H62B	108.1
C27—C26—C25	112.0 (7)	N2—C63—C68	113.2 (6)
C27—C26—H26A	109.2	N2—C63—C64	109.6 (6)
C25—C26—H26A	109.2	C68—C63—C64	109.6 (6)
C27—C26—H26B	109.2	N2—C63—H63	108.1
C25—C26—H26B	109.2	C68—C63—H63	108.1
H26A—C26—H26B	107.9	C64—C63—H63	108.1
C28—C27—C26	110.4 (6)	C63—C64—C65	111.2 (7)
C28—C27—H27A	109.6	C63—C64—H64A	109.4
C26—C27—H27A	109.6	C65—C64—H64A	109.4
C28—C27—H27B	109.6	C63—C64—H64B	109.4
C26—C27—H27B	109.6	C65—C64—H64B	109.4
H27A—C27—H27B	108.1	H64A—C64—H64B	108.0
C27—C28—C23	111.8 (6)	C66—C65—C64	108.8 (7)
C27—C28—H28A	109.2	C66—C65—H65A	109.9
C23—C28—H28A	109.2	C64—C65—H65A	109.9
C27—C28—H28B	109.2	C66—C65—H65B	109.9
C23—C28—H28B	109.2	C64—C65—H65B	109.9
H28A—C28—H28B	107.9	H65A—C65—H65B	108.3
C34—C29—C30	117.9 (5)	C65—C66—C67	112.3 (8)
C34—C29—Sn2	120.6 (4)	C65—C66—H66A	109.1
C30—C29—Sn2	121.5 (4)	C67—C66—H66A	109.1
C31—C30—C29	121.1 (6)	C65—C66—H66B	109.1
C31—C30—H30	119.5	C67—C66—H66B	109.1
C29—C30—H30	119.5	H66A—C66—H66B	107.9
C32—C31—C30	120.1 (6)	C68—C67—C66	112.9 (8)
C32—C31—H31	120.0	C68—C67—H67A	109.0
C30—C31—H31	120.0	C66—C67—H67A	109.0
C33—C32—C31	119.8 (6)	C68—C67—H67B	109.0
C33—C32—H32	120.1	C66—C67—H67B	109.0
C31—C32—H32	120.1	H67A—C67—H67B	107.8
C32—C33—C34	120.2 (6)	C63—C68—C67	111.7 (8)
C32—C33—H33	119.9	C63—C68—H68A	109.3
C34—C33—H33	119.9	C67—C68—H68A	109.3
C33—C34—C29	121.0 (6)	C63—C68—H68B	109.3
C33—C34—H34	119.5	C67—C68—H68B	109.3
C29—C34—H34	119.5	H68A—C68—H68B	107.9
			
C7—Sn1—O1—C19	59.3 (4)	C24—C23—C28—C27	55.6 (7)
C13—Sn1—O1—C19	172.4 (4)	Sn2—C23—C28—C27	−179.4 (5)
C1—Sn1—O1—C19	−68.5 (5)	C35—Sn2—C29—C34	50.7 (5)
C7—Sn1—O3—C21	177.4 (6)	C23—Sn2—C29—C34	−129.8 (4)
C13—Sn1—O3—C21	64.3 (5)	O5—Sn2—C29—C34	144.7 (5)
C1—Sn1—O3—C21	−54.8 (6)	O7—Sn2—C29—C34	−34.9 (4)
C29—Sn2—O5—C41	−44.5 (6)	C35—Sn2—C29—C30	−130.6 (5)
C35—Sn2—O5—C41	75.4 (6)	C23—Sn2—C29—C30	48.9 (5)
C23—Sn2—O5—C41	−163.7 (6)	O5—Sn2—C29—C30	−36.6 (5)
C29—Sn2—O7—C43	−66.8 (5)	O7—Sn2—C29—C30	143.8 (4)
C35—Sn2—O7—C43	173.4 (5)	C34—C29—C30—C31	−0.1 (8)
C23—Sn2—O7—C43	52.5 (5)	Sn2—C29—C30—C31	−178.8 (5)
C7—Sn1—C1—C6	27.9 (7)	C29—C30—C31—C32	0.1 (10)
C13—Sn1—C1—C6	−149.2 (5)	C30—C31—C32—C33	−1.1 (10)
O1—Sn1—C1—C6	121.7 (6)	C31—C32—C33—C34	2.1 (10)
O3—Sn1—C1—C6	−56.4 (6)	C32—C33—C34—C29	−2.1 (10)
C7—Sn1—C1—C2	−119.0 (6)	C30—C29—C34—C33	1.0 (9)
C13—Sn1—C1—C2	63.9 (7)	Sn2—C29—C34—C33	179.8 (5)
O1—Sn1—C1—C2	−25.3 (6)	C29—Sn2—C35—C36	82.8 (8)
O3—Sn1—C1—C2	156.7 (6)	C23—Sn2—C35—C36	−96.7 (8)
C6—C1—C2—C3	40.4 (10)	O5—Sn2—C35—C36	−10.7 (8)
Sn1—C1—C2—C3	−173.1 (6)	O7—Sn2—C35—C36	170.1 (8)
C1—C2—C3—C4	−42.7 (11)	C29—Sn2—C35—C40	−103.3 (8)
C2—C3—C4—C5	44.4 (12)	C23—Sn2—C35—C40	77.3 (9)
C3—C4—C5—C6	−42.6 (11)	O5—Sn2—C35—C40	163.3 (8)
C2—C1—C6—C5	−37.9 (10)	O7—Sn2—C35—C40	−16.0 (8)
Sn1—C1—C6—C5	175.1 (5)	C40—C35—C36—C37	4.9 (18)
C4—C5—C6—C1	38.4 (10)	Sn2—C35—C36—C37	179.1 (10)
C13—Sn1—C7—C12	123.5 (7)	C35—C36—C37—C38	0(2)
C1—Sn1—C7—C12	−53.6 (8)	C36—C37—C38—C39	−9(2)
O1—Sn1—C7—C12	−148.0 (7)	C37—C38—C39—C40	12 (2)
O3—Sn1—C7—C12	32.3 (7)	C36—C35—C40—C39	−1.4 (18)
C13—Sn1—C7—C8	−56.3 (6)	Sn2—C35—C40—C39	−175.7 (11)
C1—Sn1—C7—C8	126.5 (5)	C38—C39—C40—C35	−7(2)
O1—Sn1—C7—C8	32.2 (6)	Sn2—O5—C41—O6	−13.2 (11)
O3—Sn1—C7—C8	−147.5 (6)	Sn2—O5—C41—C42	168.1 (5)
C12—C7—C8—C9	−4.1 (11)	O6—C41—C42—F5	141.8 (8)
Sn1—C7—C8—C9	175.8 (5)	O5—C41—C42—F5	−39.4 (10)
C7—C8—C9—C10	3.7 (12)	O6—C41—C42—F6	34.8 (9)
C8—C9—C10—C11	−4.2 (14)	O5—C41—C42—F6	−146.4 (6)
C9—C10—C11—C12	5.2 (15)	O6—C41—C42—Cl3	−87.8 (7)
C8—C7—C12—C11	5.1 (13)	O5—C41—C42—Cl3	91.0 (6)
Sn1—C7—C12—C11	−174.8 (7)	Sn2—O7—C43—O8	−6.7 (9)
C10—C11—C12—C7	−5.8 (15)	Sn2—O7—C43—C44	175.1 (4)
C7—Sn1—C13—C14	110.5 (5)	O8—C43—C44—F8	−72.2 (9)
C1—Sn1—C13—C14	−72.0 (6)	O7—C43—C44—F8	106.1 (7)
O1—Sn1—C13—C14	19.8 (5)	O8—C43—C44—F7	172.0 (6)
O3—Sn1—C13—C14	−162.7 (5)	O7—C43—C44—F7	−9.7 (9)
C7—Sn1—C13—C18	−67.3 (5)	O8—C43—C44—Cl4	50.2 (8)
C1—Sn1—C13—C18	110.2 (5)	O7—C43—C44—Cl4	−131.5 (6)
O1—Sn1—C13—C18	−158.0 (5)	C51—N1—C45—C50	178.0 (6)
O3—Sn1—C13—C18	19.5 (5)	C51—N1—C45—C46	57.6 (10)
C18—C13—C14—C15	−0.7 (10)	N1—C45—C46—C47	−174.6 (11)
Sn1—C13—C14—C15	−178.6 (5)	C50—C45—C46—C47	65.2 (13)
C13—C14—C15—C16	0.1 (11)	C45—C46—C47—C48	−63.4 (16)
C14—C15—C16—C17	0.5 (13)	C46—C47—C48—C49	59.7 (15)
C15—C16—C17—C18	−0.3 (13)	C47—C48—C49—C50	−56.2 (14)
C16—C17—C18—C13	−0.4 (11)	N1—C45—C50—C49	−178.7 (7)
C14—C13—C18—C17	0.9 (10)	C46—C45—C50—C49	−57.3 (10)
Sn1—C13—C18—C17	178.8 (5)	C48—C49—C50—C45	52.3 (12)
Sn1—O1—C19—O2	2.6 (8)	C45—N1—C51—C56	179.2 (6)
Sn1—O1—C19—C20	−176.3 (4)	C45—N1—C51—C52	55.8 (9)
O2—C19—C20—F1	−35.6 (10)	N1—C51—C52—C53	178.2 (9)
O1—C19—C20—F1	143.4 (7)	C56—C51—C52—C53	54.5 (12)
O2—C19—C20—F2	−154.5 (7)	C51—C52—C53—C54	−55.5 (15)
O1—C19—C20—F2	24.5 (9)	C52—C53—C54—C55	54.2 (15)
O2—C19—C20—Cl1	88.8 (6)	C53—C54—C55—C56	−53.9 (13)
O1—C19—C20—Cl1	−92.2 (6)	N1—C51—C56—C55	176.8 (6)
Sn1—O3—C21—O4	−6.3 (10)	C52—C51—C56—C55	−57.4 (9)
Sn1—O3—C21—C22	175.9 (4)	C54—C55—C56—C51	58.0 (11)
O4—C21—C22—F3	24.2 (9)	C63—N2—C57—C58	60.7 (8)
O3—C21—C22—F3	−157.7 (6)	C63—N2—C57—C62	−175.7 (6)
O4—C21—C22—F4	144.4 (7)	C62—C57—C58—C59	55.6 (8)
O3—C21—C22—F4	−37.6 (8)	N2—C57—C58—C59	176.8 (6)
O4—C21—C22—Cl2	−97.4 (7)	C57—C58—C59—C60	−54.8 (9)
O3—C21—C22—Cl2	80.7 (6)	C58—C59—C60—C61	55.7 (9)
C29—Sn2—C23—C28	−150.3 (4)	C59—C60—C61—C62	−55.7 (10)
C35—Sn2—C23—C28	29.1 (5)	C58—C57—C62—C61	−57.2 (10)
O5—Sn2—C23—C28	−61.0 (4)	N2—C57—C62—C61	178.4 (7)
O7—Sn2—C23—C28	117.8 (4)	C60—C61—C62—C57	56.7 (11)
C29—Sn2—C23—C24	−25.4 (5)	C57—N2—C63—C68	54.4 (8)
C35—Sn2—C23—C24	154.0 (4)	C57—N2—C63—C64	177.1 (6)
O5—Sn2—C23—C24	63.9 (4)	N2—C63—C64—C65	175.0 (6)
O7—Sn2—C23—C24	−117.3 (4)	C68—C63—C64—C65	−60.3 (9)
C28—C23—C24—C25	−55.6 (7)	C63—C64—C65—C66	60.1 (10)
Sn2—C23—C24—C25	178.7 (4)	C64—C65—C66—C67	−55.0 (11)
C23—C24—C25—C26	55.9 (8)	C65—C66—C67—C68	51.9 (13)
C24—C25—C26—C27	−56.7 (9)	N2—C63—C68—C67	177.7 (7)
C25—C26—C27—C28	56.4 (8)	C64—C63—C68—C67	55.0 (10)
C26—C27—C28—C23	−55.5 (8)	C66—C67—C68—C63	−51.3 (13)

### Hydrogen-bond geometry (Å, °)

**Table:**

*D*—H···*A*	*D*—H	H···*A*	*D*···*A*	*D*—H···*A*
N1—H1n1···O2	0.88	1.93	2.797 (7)	166
N1—H1n2···O6^i^	0.88	1.97	2.798 (7)	155
N2—H2n2···O4	0.88	1.94	2.798 (7)	165
N2—H2n1···O8	0.88	1.94	2.759 (6)	155

Symmetry codes: (i) *x*−1, *y*, *z*.
